# Reviewed article: Comerio T, Cola JP, Mascarello KC, Sale CMM, Vasconcelos AS, Souza MC, Maciel ELN. COVID-19 vaccination status and factors associated with deaths from the disease in Vitória, Espírito Santo: cross-sectional study, 2020-2023. Epidemiol Serv Saude. 2025:34;e20240700.

**DOI:** 10.1590/S2237-96222025v34e20240700.a

**Published:** 2025-08-08

**Authors:** Samuel Lopes dos Santos

**Table:** 

Rating	Excellent	Good	Average	Below average	Poor
Interest		✓			
Quality			✓		
Originality		✓			
Overall		✓			

**Table:** 

Questionnaire	Yes	No	Not applicable
Does the manuscript contain new and significant information to justify publication?	✓		
Does the Abstract (Summary) clearly and accurately describe the content of the article?	✓		
Is the problem significant and concisely stated?	✓		
Are the methods described comprehensively?	✓		
Are the interpretations and conclusions justified by the results?	✓		
Is adequate reference made to other work in the field?	✓		

## Manuscript Structure

Length of article is: Adequate

Number of tables is: Too many

Number of figures is: Too few

Please state any conflict(s) of interest that you have in relation to the review of this paper (state “none” if this is not applicable): None

## Comments

Título: o termo “pelo agravo” apesar de claramente está associado ao COVID-19 poderá causar confusão em leitores, futuramente. (opcional o ajuste).

Objetivo: conforme.

Métodos: É necessário sanar a dúvida se foi utilizado dados obtidos diretamente dos sistemas de informação do próprio município e qual o procedimento se utilizou (Vacina e Confia). Citar se houve autorização para uso dos dados. Identificar nos preceitos éticos mesmo dispensando o TCLE, que foi resguardado os princípios gerais da LGPD (Lei geral de Proteção de Dados).

Resultados: Deixar devidamente expresso nos títulos das tabelas o valor (n) dos casos vacinados e (n) dos casos não vacinados. É importante considerar dentre cada categoria a gravidade dos diagnosticados com COVID-19 (leve, moderado ou grave).

Seria muito interessante que fosse destacado qual o tipo de imunobiológico/laboratório esteve associado aos casos de óbitos entre os vacinados.

Discussão: revisão ortográfica obrigatória. Observa-se parágrafos em que os autores expressam sua opinião, tal fato não deve ocorrer “, nosso resultado” (p 9; L2). Sugiro (no presente estudo).

Conclusão: Revisão ortográfica.

“A combinação de imunização com doses de reforço de vacinas atualizadas para variantes circulantes e intervenções não farmacológicas baseadas em evidências é fundamental para reduzir a morbimortalidade da COVID-19 e promover melhores resultados de saúde pública.”

Acredito que apesar de ser uma observação plausível os resultados do estudo em questão não fazem tal abordagem e não possui resultados que possam levar a esta inferência no final.

Sugiro ainda que, inicie o tópico de conclusão com inferência se o objetivo do estudo foi ou não alcançado, se total ou parcial. Por fim, de forma clara e direta, direcione os achados do estudo. Finalize com indicações das limitações do estudo atual e sugestões para pesquisas futuras que possam preencher as lacunas observadas ou trazer novas contribuições.

Referências: Conforme. 

